# Supplementary material for: Leopard and spotted hyena densities in the Lake Mburo National Park, southwestern Uganda
Source: PeerJ. 2022 Jan 27;10:e12307. doi: 10.7717/peerj.12307 (PMC8801179; doi:10.7717/peerj.12307)
Supplement: Supplemental Information 6 [file peerj-10-12307-s006.docx]

**Code to implement diagnostics for the winning leopard model**

Importantly, you will use this code only to generate your final posterior estimates of density, sigma etc. The code will also provide you with the ability to compare models head to head based upon their marginal likelihood (end of this document). To obtain the respective files to be able to get to this step, please use the input files provided in the secrbayes package.

**To read in your data use the following code (adapt for hyenas accordingly) – the last line of code is the model structure which you will tweak based upon which model you’d like to run – all models are provided for both species:**

Setwd("D:/Users/uqabracz/Documents/Uganda/Leopards Hyenas Mburo/Full 15 km")

statespace <- read.csv("Supporting_Information_3_Mburo_habitat.csv")

traps <- read.csv("peerj-58862-Supporting_Information_3_Mburo_traps.csv")

captures <- read.csv("peerj-58862-Supporting_Information_3_Mburo_leopard_captures.csv")

sex <- read.table("peerj-58862-Supporting_Information_3_Mburo_Leopard_sex.csv", header=TRUE, sep=";", quote="\"", stringsAsFactors=FALSE)

install.packages("doMC", repos="http://R-Forge.R-project.org")

library(doMC)

source("e2dist.R")

source("SCRi.fn.par1-QEPVIRUNGA.R")

source("scrDataWOeffort.R")

Xsex <- sex[,2]

scrMburoData <- scrData(traps=traps, captures=captures, statespace=statespace, Xsex=Xsex)

niter <- 11000

burnin <- 1000

nchains <- 2

modelno <- 1

Mburodata2 <- SCRi.fn.par1(scrMburoData, modelno=modelno, nc=nchains, ni = niter, burn = 1000, skip = 1, nz = 1500,theta=0.75,Msigma = 1, Mb = 0, Msex=1, Msexsigma = 1, Xsex = Xsex, ss.prob=NULL, coord.scale = 1000, area.per.pixel = 0.336, thinstatespace = 1, maxNN = 40, dumprate = 1000)

**The models to run for leopards are as follows:**

1) Most parameterized model – in this model theta is estimated by the model

(MSEX=1, MSEXSIGMA=1, XSEX=XSEX, THETA=NA)

Queendata <- SCRi.fn.par1(Queendata1, modelno=modelno, nc=nchains, ni = niter, burn = 5000, skip = 1, nz = 1500,theta=NA,Msigma = 1, Mb = 0, Msex=1, Msexsigma =1, Xsex = Xsex, ss.prob=NULL, coord.scale = 1000, area.per.pixel = 0.336, thinstatespace = 1, maxNN = 40, dumprate = 1000)

2) A model where Msex is not estimated but Msexsigma is – Theta is not specified

(MSEX=0, MSEXSIGMA=1, XSEX=XSEX, XEFF=XEFFORT, THETA=NA)

Queendata <- SCRi.fn.par1(Queendata1, modelno=modelno, nc=nchains, ni = niter, burn = 5000, skip = 1, nz = 1500,theta=NA,Msigma = 1, Mb = 0, Msex=0, Msexsigma = 1, Xsex = Xsex, ss.prob=NULL, coord.scale = 1000, area.per.pixel = 0.336, thinstatespace = 1, maxNN = 40, dumprate = 1000)

3) Model where theta is fixed at 0.75 and Msex is not estimated

(MSEX=0, MSEXSIGMA=1, XSEX=XSEX, XEFF=XEFFORT, THETA=0.75)

Queendata <- SCRi.fn.par1(Queendata1, modelno=modelno, nc=nchains, ni = niter, burn = 5000, skip = 1, nz = 1500,theta=0.75,Msigma = 1, Mb = 0, Msex=0, Msexsigma = 1, Xsex = Xsex, ss.prob=NULL, coord.scale = 1000, area.per.pixel = 0.336, thinstatespace = 1, maxNN = 40, dumprate = 1000)

4) Theta is fixed at 0.75 and Msex is estimated

(MSEX=1, MSEXSIGMA=1, XSEX=XSEX, XEFF=XEFFORT, THETA=0.75)

Queendata <- SCRi.fn.par1(Queendata1, modelno=modelno, nc=nchains, ni = niter, burn = 5000, skip = 1, nz = 1500,theta=0.75,Msigma = 1, Mb = 0, Msex=1, Msexsigma = 1, Xsex = Xsex, ss.prob=NULL, coord.scale = 1000, area.per.pixel = 0.336, thinstatespace = 1, maxNN = 40, dumprate = 1000)

5) Msex is estimated but Msexsigma is not and theta is fixed at 0.75

(MSEX=1, MSEXSIGMA=0, XSEX=XSEX, XEFF=XEFFORT, THETA=0.75)

Queendata <- SCRi.fn.par1(Queendata1, modelno=modelno, nc=nchains, ni = niter, burn = 5000, skip = 1, nz = 1500,theta=0.75,Msigma = 1, Mb = 0, Msex=1, Msexsigma =0, Xsex = Xsex, ss.prob=NULL, coord.scale = 1000, area.per.pixel = 0.336, thinstatespace = 1, maxNN = 40, dumprate = 1000)

6) Most parameterized model but theta is set to 1 unlike in the very first model where theta is estimated by the model

(MSEX=1, MSEXSIGMA=1, XSEX=XSEX, THETA=NA)

Mburodata <- SCRi.fn.par1(Queendata1, modelno=modelno, nc=nchains, ni = niter, burn = 5000, skip = 1, nz = 1500,theta=1,Msigma = 1, Mb = 0, Msex=1, Msexsigma =1, Xsex = Xsex, ss.prob=NULL, coord.scale = 1000, area.per.pixel = 0.336, thinstatespace = 1, maxNN = 40, dumprate = 1000)

For hyenas, these are the two models we ran:

1) Theta set to 1

(MSEX=0, MSEXSIGMA=0, XSEX=NULL, THETA=1)

Mburodata1 <- SCRi.fn.par1(scrMburoData, modelno=modelno, nc=nchains, ni = niter, burn = 1000, skip = 1, nz = 1500,theta=1,Msigma = 1, Mb = 0, Msex=0, Msexsigma = 0, Xsex = NULL, ss.prob=NULL, coord.scale = 1000, area.per.pixel = 0.336, thinstatespace = 1, maxNN = 40, dumprate = 1000)

2) A model where Theta is estimated by the model

(MSEX=0, MSEXSIGMA=1, XSEX=XSEX, XEFF=XEFFORT, THETA=NA)

Mburodata2 <- SCRi.fn.par1(scrMburoData, modelno=modelno, nc=nchains, ni = niter, burn = 1000, skip = 1, nz = 1500,theta=NA,Msigma = 1, Mb = 0, Msex=0, Msexsigma = 0, Xsex = NULL, ss.prob=NULL, coord.scale = 1000, area.per.pixel = 0.336, thinstatespace = 1, maxNN = 40, dumprate = 1000)

**### Code to do MCMC diagnostics and calculate posterior HPDs of estimates in Bayesian SECR**

**### For QEP-Virunga-SearchEncounter-Analysis**

## Call coda package (for MCMC diagnostics) and mcmcse package (to compute Monte Carlo error)

library(coda)

library(mcmcse)

library(parallel)

### Obtain all the MCMC histories. These directory structures have to be replaced by the ones obtained after running the analysis ###

histCH1 <- read.csv("QEP-VIRUNGA-mcmchist_190304_115907CH1.csv")

gdataCH1 <- read.csv("gofdata_190304_115907CH1.csv")

gnewCH1 <- read.csv("gofnew_190304_115907CH1.csv")

### Activate these only on good computers ####

AcCentresCH1 <- read.csv("AcCentres_190304_115907CH1.csv")

RealIndividualsCH1 <-read.csv("RealIndividuals_190304_115907CH1.csv")

histCH2 <- read.csv("QEP-VIRUNGA-mcmchist_190304_115953CH1.csv")

gdataCH2 <- read.csv("gofdata_190304_115953CH1.csv")

gnewCH2 <- read.csv("gofnew_190304_115953CH1.csv")

AcCentresCH2 <- read.csv("AcCentres_190304_115953CH1.csv")

RealIndividualsCH2 <- read.csv("RealIndividuals_190304_115953CH1.csv")

histCH3 <- read.csv("QEP-VIRUNGA-mcmchist_190304_120140CH1.csv")

gdataCH3 <- read.csv("gofdata_190304_120140CH1.csv")

gnewCH3 <- read.csv("gofnew_190304_120140CH1.csv")

AcCentresCH3 <- read.csv("AcCentres_190304_120140CH1.csv")

RealIndividualsCH3 <- read.csv("RealIndividuals_190304_120140CH1.csv")

histCH4 <- read.csv("QEP-VIRUNGA-mcmchist_190304_121712CH1.csv")

gdataCH4 <- read.csv("gofdata_190304_121712CH1.csv")

gnewCH4 <- read.csv("gofnew_190304_121712CH1.csv")

AcCentresCH4 <- read.csv("AcCentres_190304_121712CH1.csv")

RealIndividualsCH4 <- read.csv("RealIndividuals_190304_121712CH1.csv")

#### Create MCMC objects ####

histCH1mcmc <- as.mcmc(histCH1)

histCH2mcmc <- as.mcmc(histCH2)

histCH3mcmc <- as.mcmc(histCH3)

histCH4mcmc <- as.mcmc(histCH4)

## Remove beta.behave column, X column(iter no) and Density(for a strange reason gives an error) and set start and end for extended burnin

start<-2001

end<-10000

histCH1mcmc <- window(histCH1mcmc[,c(-1,-7,-13)], start,end)

histCH2mcmc <- window(histCH2mcmc[,c(-1,-7,-13)], start,end)

histCH3mcmc <- window(histCH3mcmc[,c(-1,-7,-13)], start,end)

histCH4mcmc <- window(histCH4mcmc[,c(-1,-7,-13)], start,end)

### Combine chain outputs ###

combinedHist <- rbind(histCH1mcmc, histCH2mcmc, histCH3mcmc, histCH4mcmc)

chainList <- list(histCH1mcmc, histCH2mcmc, histCH3mcmc, histCH4mcmc)

## MCMC diagnostics ##

## Multi-chain convergence check using Gelman-Rubin diagnostic

gelmandiag <- gelman.diag(chainList, confidence=FALSE, transform=FALSE, autoburnin=FALSE, multivariate=FALSE)

## Single chain convergence check using Geweke diagnostic (optional)

gewekediag <- geweke.diag(histCH1mcmc)

#### Report MCMC diagnostic results. For Geweke we want the magnitude (-ve or +ve) for each parameter to be less than 1.64. For Gelman-Rubin we want Potential Shrink Reduction Factor to be less than 1.2 (1.1 or lower for more defensible runs) for each parameter.

gelmandiag

gewekediag

### Summary results. Look for how different median is to the mean. This indicates nature of the posterior distribution. Ideally we would like them to be nearly the same. But OK otherwise too.

mean.model1 <- apply(combinedHist,2,mean)

## Obtain mean of the estimates with the Monte Carlo error

mean.model1 <- mcse.mat(combinedHist, method="bm", g=NULL)

sd.model1 <- apply(combinedHist,2,sd)

mean.model1

sd.model1

## Highest posterior density intervals for one of the chains # This piece of code is taken from SPACECAP version 1.1.0 (Gopalaswamy et al. 2015) ##

HPDinterval(histCH1mcmc)

#### Goodness-of-fit statistics ####

### Obtain all the gof statistics ###

### Combine gdata and gnew ###

gdatacombined <- rbind(gdataCH1, gdataCH2, gdataCH3, gdataCH4)

gnewcombined <- rbind(gnewCH1, gnewCH2, gnewCH3, gnewCH4)

## Bayesian p-value calculation ##

BayesPval <- mean(gdatacombined[,2]>gnewcombined[,2])

BayesPval

## Generate pair-wise plots. This will be useful for assessing estimation covariances and parameter redundancies (if any) owing to poor sample sizes ##

pairs(combinedHist, gap=0, pch=".")

### Generate pixel-specific density estimates ###

## Combine activity centres and real individuals file into a combined history ##

AcCentresCombined <- rbind(AcCentresCH1, AcCentresCH2, AcCentresCH3, AcCentresCH4)

RealIndividualsCombined <- rbind(RealIndividualsCH1, RealIndividualsCH2, RealIndividualsCH3, RealIndividualsCH4)

## Obtain the unscaled statespace (any one chain is sufficient as it comes from input data) ##

SSunscaledCH <- read.csv("SSunscaled_190304_115907CH1.csv")

nG <- nrow(SSunscaledCH)

# Set pixel ID of home range centers for phantom animals to zero

indlocsCH1 <- AcCentresCH1 * RealIndividualsCH1

indlocsCH2 <- AcCentresCH2 * RealIndividualsCH2

indlocsCH3 <- AcCentresCH3 * RealIndividualsCH3

indlocsCH4 <- AcCentresCH4 * RealIndividualsCH4

indlocs <- rbind(indlocsCH1, indlocsCH2, indlocsCH3, indlocsCH4)

indlocnum <- data.matrix(indlocs)

# Count the proportion of times each pixel was a home range centre,

# convert to animals per sq km (here 1 sq km was input data for elephant analysis - so change accordingly)

densVec <- tabulate(indlocnum, nbins=nG) / nrow(indlocs) / 0.158

dirMain <- "D:/Users/uqabracz/Documents/Uganda/Leopards Hyenas Mburo/Leopards/X25km/Diagnostics"

setwd(dirMain)

GEC_Loango_CAMTRAP_SS <- read.csv("Mburo habitat.csv")

pixelDensity <- GEC_Loango_CAMTRAP_SS

pixelDensity$`Pixel Density` <- GEC_Loango_CAMTRAP_SS[, 3]

pixelDensity$`Pixel Density`[GEC_Loango_CAMTRAP_SS[, 3] > 0] <- densVec

# Generate csv file for pixel densities #

nameoffile3 = paste(dirMain,"/GEC_Loango_CAMTRAP_PixelDens.csv", sep="")

write.csv(pixelDensity, file=nameoffile3)

# This part is to obtain posterior standard deviations on pixel-specific densities #

# Create an abundance matrix of dimension no. of iterations x total number of grid cells #

abundMatrix <- matrix(data=NA, nrow=nrow(indlocs), ncol=nG)

# Fill up this matrix with abundance counts for each iteration #

for (i in 1:nrow(indlocs)){

abundVecTemp <- tabulate(indlocnum[i,], nbins=nG)

abundMatrix[i,] <- abundVecTemp

}

# This part is meant to compute abundances for sub-regions #

# Enter the sequence of grid cell numbers for analysis. This will be a selection of numbers between 1 to nG corresponding to which cells are being analysed. For the entire study area this can simply be 1:nG. In the example below, it indicates that only grid cells 1,3,5,7 are chosen for reporting. This will be according to the sub-region chosen #

gridVec <- c(1,3,5,7, 15, 19)

# Obtain total abundance counts for grid cells referenced by gridVec for each iteration #

abundVecTotal <- rowSums(abundMatrix[,gridVec])

# Obtain posterior mean and standard deviations of the sub-region

meanAbund <- mean(abundVecTotal)

sdAbund <- sd(abundVecTotal)

**Results Global 25km**

**Model selection:**

##### R code snippet to estimate the marginal likelihood and its associated standard deviation from LogLikelihood outputs in SECR #####

rm(list = ls())

options(digits = 8)

source('BMSE.utility.functions.1.R')

start.time = Sys.time()

ts = format(Sys.time(), "%d%m%y_%H%M%S")

#=================================================

# Harmonic mean estimator of marginal likelihood

#=================================================

# g(mu, L) = pi(mu, L) i.e Takng prior of (mu, L) as the tuning density of (mu, L)

#loglik.chain =  unlist(read.csv(paste0(folderpath, '/markovchain.loglikelihood.txt', sep = ''), sep = ',', header = T))[(burnin + 1):ndraws]

#if(model == 1 | model  == 2)

#      {

#        logfactor.sex = log(post.theta^(post.z[,1:numl]*post.sex[,1:numl]))  + log((1 - post.theta)^(1 - post.z[,1:numl]*post.sex[,1:numl])) # tot.length x numl

#        loglik.chain = loglik.chain + rowSums(logfactor.sex)  # tot.length x 1

#      }

###### Read the LogLikelihood vector from the output file ######

logCH1 <- read.csv("LogLikelihood_190304_115907CH1.csv")

logCH2 <- read.csv("LogLikelihood_190304_115953CH1.csv")

logCH3 <- read.csv("LogLikelihood_190304_120140CH1.csv")

logCH4 <- read.csv("LogLikelihood_190304_121712CH1.csv")

combinedlike <- rbind(logCH1, logCH2, logCH3, logCH4)

loglik.file <- combinedlike

loglik.chain <- loglik.file[,2]

logh.chain = - loglik.chain # -loglik.zx0s.chain

C = mean(logh.chain)

[logmarglik.hm](http://logmarglik.hm/) = gdmean3(logh.chain)

tot.length = length(loglik.chain)

sd.mhm = gdsd(logh.chain)

cat('Log of the estimated Marginal Likelihood using HM method =', [logmarglik.hm](http://logmarglik.hm/))
